# Whole-genome sequencing and annotation of six metallotolerant bacterial isolates recovered from a retention pond at the Rochester Institute of Technology, New York

**DOI:** 10.1128/mra.00273-26

**Published:** 2026-05-28

**Authors:** Danae K. R. Bardaji, Girish Kumar, Azahria Williams, Gabriella Green, Moriah Wilson, Madelyn Wilson, Olivia Harper, Gabriella Fedus, Aidan Liddell, Pranav Bhagavatula, André O. Hudson

**Affiliations:** 1 Thomas H. Gosnell School of Life Sciences, Rochester Institute of Technology, Rochester, New York, USA; 2 Rochester Prep High School, Rochester, New York, USA; Fluxus Inc., Sunnyvale, California, USA

**Keywords:** metallotolerant, copper, zinc

## Abstract

We report draft genome sequences of six metallotolerant bacterial isolates recovered from copper and zinc supplemented media inoculated with environmental samples collected from a retention pond located on the campus of the Rochester Institute of Technology, New York. These genomes provide resources for investigating environmental adaptation and genetic determinants associated with metal tolerance in freshwater bacteria.

## ANNOUNCEMENT

Heavy metals such as copper and zinc are common environmental contaminants derived from natural geological processes and anthropogenic activities (1, 2). Elevated metal concentrations can exert selective pressure on microbial communities, enriching bacteria capable of tolerating metal stress (3). Freshwater environments, therefore, represent important ecological niches for studying microbial adaptation and environmental resilience at the genomic level (4).

Water and sediment samples were collected from J-lot Pond located on the campus of the Rochester Institute of Technology (RIT), Rochester, New York, USA (43.0840°N, 77.6746°W). Samples were transferred to sterile 50-mL conical tubes and enriched in Reasoner’s 2A (R2A) broth for 7 days at 30°C. Serial dilutions (10⁻¹–10⁻⁵) were prepared and plated on R2A medium supplemented with CuSO₄ or ZnSO₄ at final concentrations of 1 mM, 3 mM, and 5 mM. Plates were incubated at 30°C for 24 h. Colonies exhibiting growth at the highest metal concentrations were streaked for purity on supplemented and non-supplemented agar. Pure colonies were grown overnight in R2A broth at 30°C prior to DNA extraction.

Six isolates were chosen for whole-genome sequencing according to their color, shape, and morphology. *Serratia ureilytica* (Sample 1) and *Bacillus cereus* (Sample 2) were isolated from agar plates containing 5 mM CuSO₄. *Serratia nematodiphila* (Sample 6) and *Stenotrophomonas africana* (Sample 8) were isolated from 5 mM ZnSO₄-supplemented agar plates. *Klebsiella variicola* (Sample 9) and *Chryseobacterium candidae* (Sample 11) were recovered from 3 mM CuSO₄- and ZnSO₄-supplemented plates, respectively.

Genomic DNA was extracted using GenElute Bacterial Genomic DNA isolation kit (Sigma-Aldrich, USA) according to the manufacturer’s protocol. DNA concentrations were measured using a Qubit 4.0 fluorometer with the 1 × dsDNA High Sensitivity Kit (Life Technologies, MD). Sequencing libraries were prepared using the Nextera XT Library Preparation Kit (Illumina Inc., USA) and sequenced on an Illumina NextSeq 2000 platform (San Diego, CA, USA) using a P1 kit (2 × 300 cycles). Sequencing generated between 12,853,754 and 39,536,190 paired-end reads per isolate (Table 1).

**TABLE 1:** Sequencing, assembly, annotation, and accession information for six metallotolerant bacterial isolates recovered from J-lot Pond (43.0840°N, 77.6746°W), Rochester, NY

Species	BestMatch Type-strain (Accession)	Sample #	ANI (%)	Raw reads	Total nucleotides (bp)	SRA accession	Assembly accession	Assembly size (bp)	Coverage (×)	Contigs (*n*)	N50 (bp)	GC (%)	Genes	rRNA	tRNA
*Serratia ureilytica*	*Serratia ureilytica* (GCA_013375155.1)	1	99.02	15,925,836	4,681,278,728	SRR37101620	GCA_055524 495.1	5,202,985	919.70	40	665,428	59.54	4,995	14	83
*Bacillus cereus*	*Bacillus cereus* ATCC 14579 (GCA_045287585.1)	2	97.19	39,536,190	11,388,869,192	SRR37101619	GCA_055524 475.1	5,815,620	2,148.84	65	497,453	35.01	5,962	21	95
*Serratia nematodiphila*	*Serratia nemato-* *diphila* (GCA_900101535.1)	6	98.30	15,543,164	4,543,845,388	SRR37101617	GCA_055524 435.1	5,245,269	853.28	34	577,886	59.50	5,033	13	82
*Stenotrophomonas africana*	*Stenotrophomonas africana* (GCA_013004645.1)	8	97.81	12,853,754	3,799,478,019	SRR37101616	GCA_055524 455.1	4,482,150	796.68	36	211,334	66.50	4,122	6	67
*Klebsiella variicola*	*Klebsiella variicola* (GCA_000828055.2)	9	99.14	13,436,068	3,955,201,809	SRR37101615	GCA_055524 415.1	5,825,342	719.13	101	364,864	57.05	5,849	16	81
*Chryseobacterium candidae*	*Chryseobacterium candidae* (GCA_004916905.1)	11	98.82	20,940,920	6,131,053,179	SRR37101614	GCA_055524 395.1	4,674,566	1,123.86	93	198,734	36.44	4,217	10	74

Default parameters were used for all software unless otherwise noted. Raw reads were quality filtered (*Q* ≥ 30), and adapter sequences were trimmed using fastp version 0.24.1 (5). *De novo* genome assemblies were generated using SPAdes version 4.2.0 (6). Species identification was performed using GTDB-Tk version 2.5.2 (7), which assigned isolates with average nucleotide identity (ANI) values ranging from 97.37% to 99.01%. Genome annotation was performed using the NCBI Prokaryotic Genome Annotation Pipeline (PGAP) version 2025-05-06.build7983 (8).

**Fig 1:**
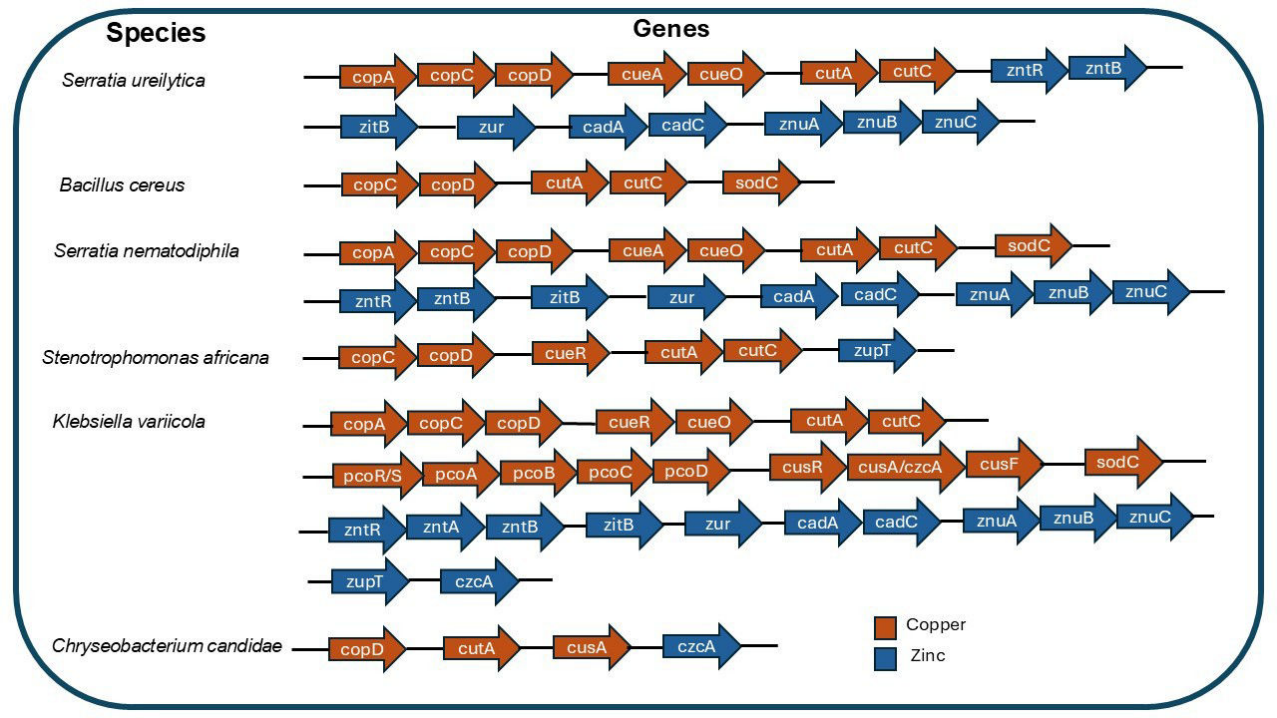
Genomic distribution of genes involved in the responses to copper (Cu) and zinc (Zn) across six bacterial species identified using the Prokaryotic Genome Annotation Pipeline (PGAP). Copper resistance genes include efflux pumps and transporters such as, copA, copC, copD, cueA, and cusA/F; regulators, cueR, pcoR/S, and pcoA/B/C/D; and detoxification or tolerance genes including cueO, cutA, cutC, and sodC. Zinc resistance and homeostasis genes encompass efflux systems such as zntA, zntB, zitB, and czcA; high-affinity uptake components znuA, znuB, znuC, and zupT; and regulatory elements including zntR, zur, cadA, and cadC. The linear arrangement of these arrows is for comparative visualization of the genetic repertoire only; the genes displayed for each species do not necessarily represent a single contiguous operon and are not necessarily proximal to one another on the bacterial genomes.

Table 1 summarizes assembly statistics, annotation, and accession numbers. The PGAP analysis identified various genes involved in maintaining cellular homeostasis under heavy metal stress. These include genes responsible for metal regulation, detoxification, and active efflux to help reduce the toxicity caused by elevated levels of metals (9–12) (Fig. 1). The sequencing and annotation data generated in this study have the potential to facilitate future studies aimed at identifying genetic determinants responsible for metal tolerance and metal and other adaptive traits in freshwater bacteria.

## Data Availability

The raw sequencing data have been deposited in the NCBI Sequence Read Archive (SRA) under BioProject accession no. PRJNA1418155. Corresponding BioSample accession nos. are SAMN55038010, SAMN55038011, SAMN55038013, SAMN55038014, SAMN55038015, and SAMN55038016.
